# Diagnostic Excellence, Imaging Stewardship, and Re-establishing the Value Proposition in Radiology: A Look Back and a Way Forward

**DOI:** 10.7759/cureus.103537

**Published:** 2026-02-13

**Authors:** Stephen Waite, Adrian Brady, Mark Graber, John D Banja, Brian Sheppard, Michael A Bruno

**Affiliations:** 1 Department of Radiology, State University of New York Downstate Health Sciences University, Brooklyn, USA; 2 Department of Radiology, University College Cork, Cork, IRL; 3 Department of Medicine, State University of New York, New York, USA; 4 Department of Rehabilitation Medicine and Center for Ethics, Emory University, Atlanta, USA; 5 School of Law, Seton Hall University, Newark, USA; 6 Department of Radiology, Penn State, Hershey, USA

**Keywords:** diagnostic excellence, diagnostic uncertainty, incidental findings, low-value imaging, overutilization

## Abstract

Historically, imaging was used to test specific clinical hypotheses and reduce diagnostic uncertainty by clarifying, narrowing, and prioritizing differential diagnoses. Advanced imaging, such as CT and MRI, was used judiciously owing to concerns about high costs and, in the case of CT scanning, the need to limit patients' exposure to ionizing radiation. But times have changed: the threshold for ordering imaging tests is now substantially lower, and as a result, a high - and growing - volume of low-value imaging is performed, often without any particular clinical or diagnostic hypothesis in mind. Several factors are contributing to this excess demand. Perhaps chief among them is a psychological discomfort with diagnostic uncertainty and a higher tolerance for low-value imaging. Other factors include perceived medicolegal risk and the widespread expectation that physicians exhaustively investigate all incidental findings. These factors are effectively changing the role of diagnostic imaging in ways that far exceed its original mandate of answering specific clinical questions. In this manuscript, we discuss these factors, review the value proposition of imaging, and attempt to place its use in the context of diagnostic excellence. We also discuss methods to curb increasing overutilization.

## Introduction and background

It has been more than a century since the German physicist Wilhelm Conrad Roentgen described the seemingly magical ability of X-rays to image human bones and organs [1]. Shortly thereafter, imaging became a standard method of making medical diagnoses. Indeed, although many diagnoses can still be confidently made from a history and physical examination alone, medical diagnosis in the 21st century is inextricably dependent upon both laboratory testing and diagnostic imaging. Over a century later, all developed countries offer advanced imaging technology as an indispensable tool for surgical and medical diagnosis. Although radiologists still perform the critical job of image interpretation to reduce clinical diagnostic uncertainty, their role (and the role of imaging writ large) has evolved substantially. Here, we examine the major factors that have contributed to this evolution, their potential impact on healthcare quality, and the evolving value proposition of imaging in healthcare.

## Review

Creating value with diagnostic radiology

**Figure 1:**
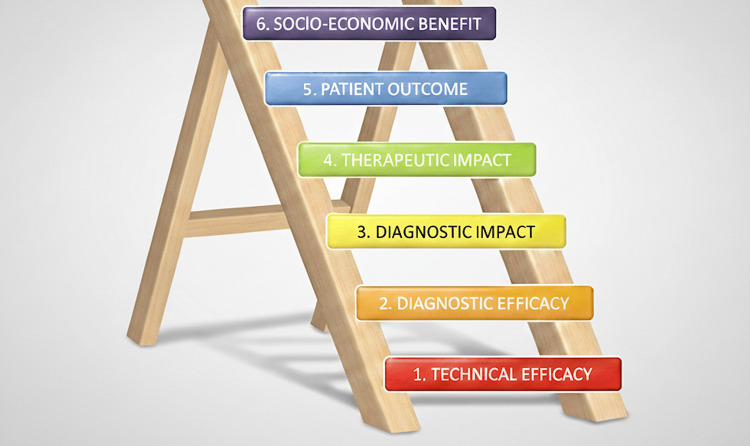
The Fryback and Thornbury hierarchical model of efficacy in radiologic imaging. This graphic was derived utilizing data from [2]. Efficacy at each lower level in this hierarchy is necessary but not sufficient to assure efficacy at higher levels. Level 1. Technical Efficacy. Technical efficacy refers to the ability to produce a reliable image. It is usually measured through physical characteristics such as spatial resolution, contrast-to-noise ratio, and sampling frequency. Level 2. Diagnostic Accuracy. Does the diagnostic study reduce uncertainty? Example measures include diagnostic accuracy, positive and negative predictive value, sensitivity and specificity, and area under the receiver operating characteristic (ROC) curve. Level 3. Diagnostic Impact. Does the reduction in diagnostic uncertainty provided by the diagnostic study change the clinical impression? Example measures include post-test change in diagnosis and post-test reduction in additional diagnostic tests. Level 4. Therapeutic Impact. Does the diagnostic study result in a change in therapy? Example measures include post-test change in the type, dose, or frequency of therapeutic intervention. Level 4 is the last level, whereby attainment is necessary but not sufficient for the creation of high-value care from the perspective of a patient receiving a diagnostic test. Level 5. Patient Outcome. Does the diagnostic study create benefit for the patient? Example measures include harm reduction (e.g., pain, anxiety, financial toxicity, morbidity, mortality), benefit increase (e.g., life expectancy, quality of life, avoiding additional tests and interventions), and improvement to the cost-to-benefit ratio. Level 5 (patient outcome) is the first level at which there is potential to realize high-value care from the patient's perspective. Level 6. Socioeconomic Benefit. Does the diagnostic study create benefit for society? Examples of measures are similar to those at the patient level (Level 5), but at the population scale. Image credits: Stephen Waite.

In 1991, Fryback and Thornbury developed a hierarchical model for assessing efficacy in radiologic imaging, with technical efficacy at the lowest level and societal impact at the highest level (Figure 1) [2]. Efficacy in this context refers to the benefit from technology applied under ideal circumstances. It is generally accepted that high-value care is only achievable at the higher levels of the pyramid [3]. Unfortunately, radiologic research rarely rises above its lower levels (i.e., technical efficacy and diagnostic accuracy) [3]. As Fletcher et al. note, "In diagnostic imaging, the accuracy of an imaging test is usually compared with established reference standards (e.g., histopathologic examination) and generally serves as a suitable surrogate for patient outcomes, as treatment benefit and efficacy is *assumed*." [4] This underlying assumption is, however, often invalid, as improved diagnostic accuracy does not necessarily result in improved patient outcomes.

Although there are exceptions, such as lung cancer screening, which is associated with decreased mortality [5], most of the radiologic literature fails to demonstrate how, or that, imaging either creates long-term and system-wide cost savings or improved patient outcomes [3].

"Value" in medicine is commonly defined as clinical outcome (benefit) divided by incremental cost [6]. High-value imaging is generally considered to be imaging supported by the use of evidence-based criteria [7] and necessitates that a test (1) be technically effective, (2) reduce diagnostic uncertainty, (3) influence treatment decisions, (4) result in effective treatment, (5) improve clinical outcomes, and (6) be of low cost relative to the clinical outcome achieved [2]. Alternatively, low-value imaging is operationally defined as imaging associated with small, neutral, or even harmful clinical outcomes at proportionately high cost [3]. As such, a non-indicated but high-quality MRI for back pain provides less value than a poor-quality head CT in a patient at high risk of intracranial hemorrhage because a CT in the latter context is more likely to be therapeutically valuable (i.e., it is more likely to demonstrate findings that influence treatment) [3].

Inappropriate and low-value imaging

Over 90 million CT scans were performed in the US in 2023 [8]. An inherent assumption driving this preponderance of imaging is that imaging generally, if not always, adds value to the diagnostic process. Imaging can rule out "worst-case scenarios," clarify diagnostic possibilities, and direct management. In short, imaging can reduce clinical uncertainty, mitigate risk, and expedite an accurate diagnosis. In many cases, a diagnosis can be established based on imaging without waiting for the disease to manifest at a later stage, when it may be less treatable. However, it is not always true that earlier detection improves health outcomes. On the contrary, well-meaning efforts to enhance operational efficiency and enable early disease detection have led to a large and growing fraction of modern imaging that does not meaningfully benefit the patient and, instead, can result in patient harm.

Rather than adding value to the diagnostic process, low-value imaging, in concert with overutilization, overdiagnosis, and overtreatment, can, perhaps ironically, result in nonexistent or even adverse health outcomes relative to cost. It is worth noting that 'cost' is a capacious concept, including, as it does, not only the monetary expenditures borne by the patient, provider, or insurer, but also the opportunity costs resulting from diminution in the capacity of providers to provide the same-level quality care to other patients in need of care. As modern radiologists operate at or near their maximum capacity, increased volumes driven by low-value imaging can effectively reduce a radiologist’s available time to spend on other patients’ examinations, potentially leading to substandard interpretations and worse outcomes [9]. Radiologists are both consciously and unconsciously aware of this fact, and low-value imaging has been cited as a factor influencing radiologist burnout, longer turnaround times, error rates, physician turnover, and early retirements, all of which contribute to worsening physician shortages and increased health care costs [10].

Diagnostic stewardship: the CDC Diagnostic Excellence Initiative (DxEx)

**Figure 2:**
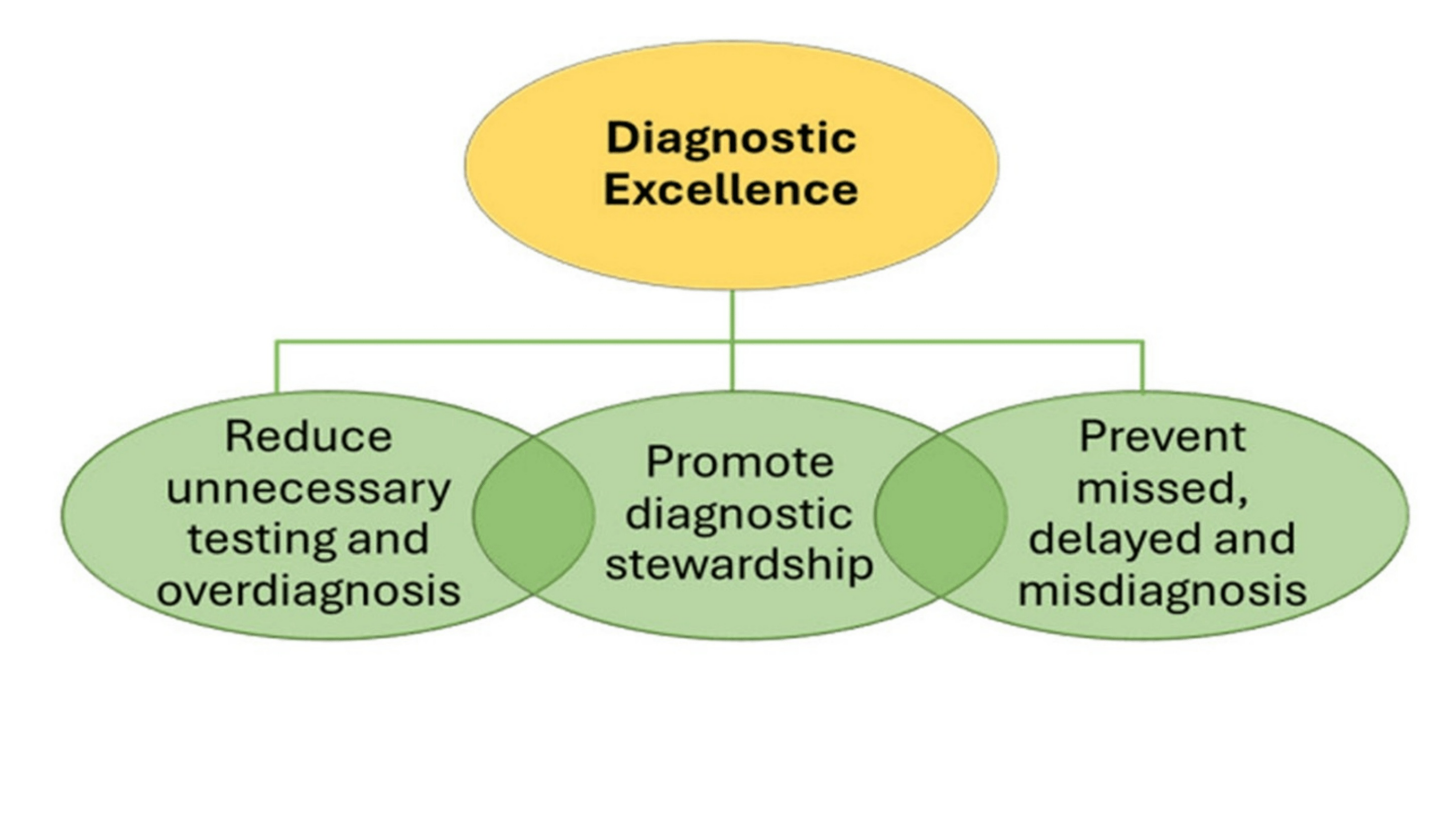
The core components of diagnostic excellence. Components of diagnostic excellence include the reduction of unnecessary testing and the promotion of diagnostic stewardship. Reprinted from [11] with permission. Note that the use of this diagram does not constitute its endorsement or recommendation by the US Government, Department of Health and Human Services, or Centers for Disease Control and Prevention. This figure is available on the agency website for no charge.

Acknowledging these problems, in 2025, the US Centers for Disease Control and Prevention (CDC) published a statement entitled "Core Elements of Hospital Diagnostic Excellence (DxEx)" as part of a national initiative to '*prevent missed, delayed, and incorrect diagnoses while reducing unnecessary testing and overdiagnosis*' [11,12]. DxEx practices note that a core component of 'diagnostic excellence' is diagnostic stewardship - the 'application of specific and evidence-based actions' during patient care, including the utilization of appropriateness criteria when performing advanced imaging (Figure 2) [11].

To achieve diagnostic excellence, physicians are admonished to avoid testing that is unlikely to benefit patients and which may instead lead to waste, inefficiency, and even harm. Noting the importance of multidisciplinary expertise, they note that radiologists can 'guide the proper use and performance of radiological imaging' [11]. As such, this CDC guidance effectively constitutes a federal mandate to reduce low-value imaging.

Drivers of low-value imaging utilization

Overutilization and Low-Value Care

**Figure 3:**
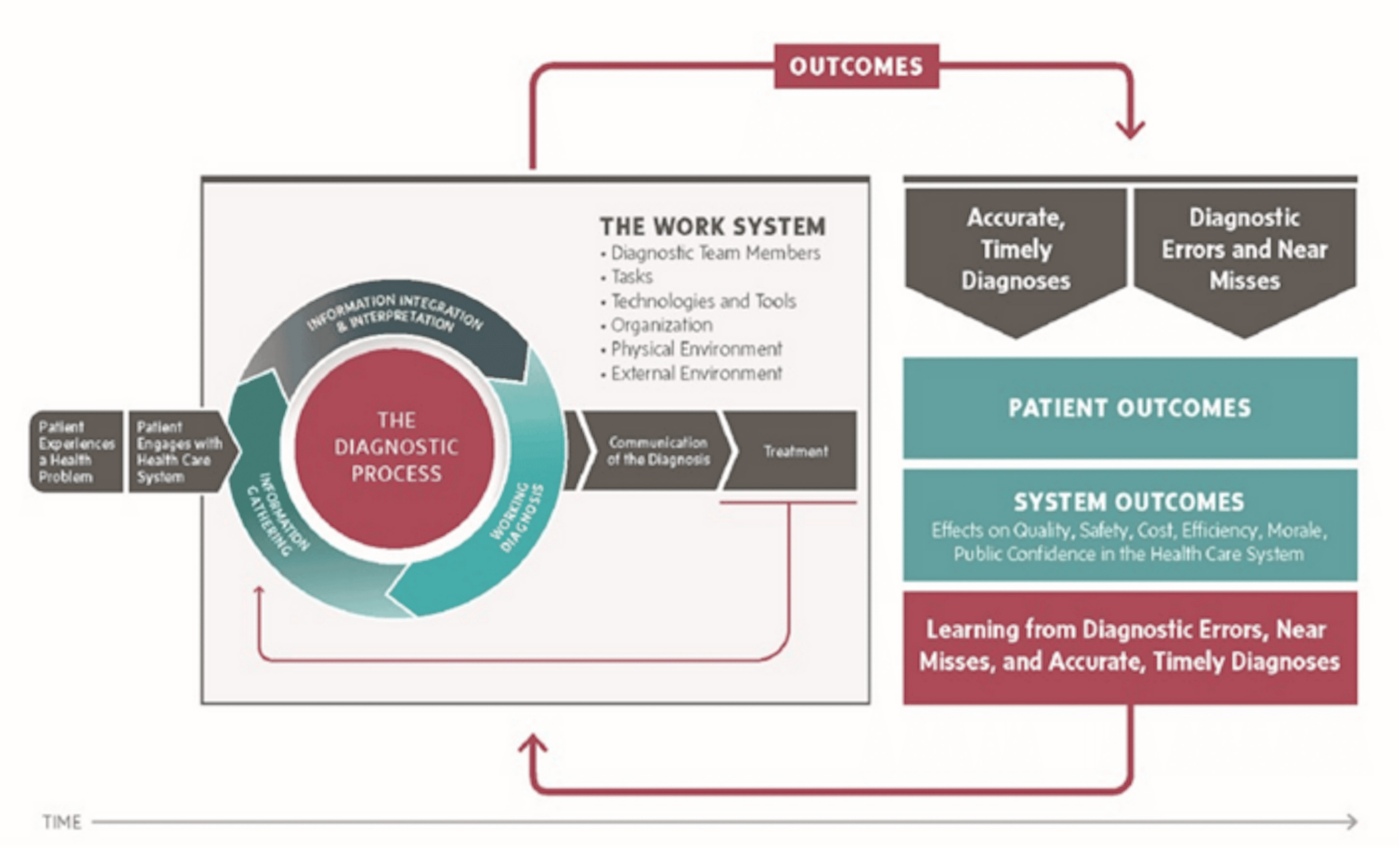
National Academy of Medicine conceptualization of the diagnostic process. The diagnostic process extends from patient presentation to treatment and outcome measurements. The goal of information gathering (including medical imaging) is to reduce diagnostic uncertainty and narrow diagnostic possibilities (the working diagnosis). Reprinted with permission from [14].

Overutilization can be defined as "an intervention in which evidence suggests it confers no or very little benefit for patients, or risk of harm exceeds probable benefit" [13]. As per the National Academy of Medicine's conceptualization of the diagnostic process, medical imaging is considered part of the information-gathering phase, and its function is to narrow diagnostic possibilities (Figure 3) [14]. As such, imaging that does not narrow possibilities, improve outcomes, or that is overly expensive relative to the clinical value it provides is considered low-value [15]. Not only does low-value imaging fail to improve individual outcomes, but it also creates post-diagnosis risks of harm for patients, including increased medical expenses and debt, radiation exposure, contrast media-related complications, emotional harm, incidental findings, false positives/negatives, overdiagnosis, overtreatment, and treatment-related complications [15].

Alarmingly, current estimates are that 20-50% of all imaging procedures are unnecessary [16,17]. Lehnert and Bree found that 26% of elective outpatient imaging examinations ordered at an academic hospital did not meet the criteria for "appropriateness" under evidence-based best-practice guidelines. These examinations were 3.5 times more likely to be negative compared with tests considered appropriate, demonstrating the relatively low value of imaging this cohort [18].

Exemplifying the problem of overutilization, a recent retrospective cohort analysis of adult patients found that 7.6% of thyroid ultrasounds performed at the Mayo Clinic from 2017 to 2021 were inappropriate [19]. Noting that this percentage was at the low end of estimates, these studies are nonetheless associated with a lower likelihood of actionable thyroid nodules, biopsy, and thyroid cancer [19]. In addition, thyroid cancers diagnosed with ultrasounds that are inappropriately ordered are usually smaller than those diagnosed with ultrasounds ordered as per established clinical guidelines [19,20]. Unfortunately (and sometimes tragically), the detection of smaller, potentially indolent lesions often leads to unnecessary and possibly harmful interventions, causing both patient anxiety and financial strain.

Discomfort With Uncertainty

Many low-value studies are ordered to reduce anxiety and discomfort among ordering clinicians [21-23]. Not surprisingly, ordering providers who are more psychologically intolerant of uncertainty tend to prescribe a larger fraction of low-value diagnostic tests [24]. For example, Hong et al. found that physicians who order very high rates of low-value imaging for back pain also have increased odds of ordering low-value imaging for headaches, suggesting a root, provider-level discomfort with uncertainty [25].

Sir William Osler, one of the founding faculty of Johns Hopkins Hospital in the late 19th century, noted that uncertainty was an integral and inherent part of medical practice, stating, "errors in judgment are inevitable in the practicing of an art which consists largely of balancing probabilities" [26]. In a paper discussing excessive testing in 1989, Dr. Jerome Kassirer echoed this sentiment, stating, "Absolute certainty in diagnosis is unattainable, no matter how much information we gather, how many observations we make, or how many tests we perform… Our task is not to attain certainty, but rather to reduce the level of diagnostic uncertainty enough to make optimal therapeutic decisions" [27]. Kassirer, astutely and presciently, foresaw the dangers of unrestricted testing, including its deleterious effects on healthcare costs, its inherent risks to the patient, and the likelihood that it would overwhelm laboratory and radiology resources [27].

Diagnostic uncertainty must be actively managed, as clinical care is inherently uncertain because scientific facts can only be applied imperfectly to the unique context of an individual patient’s life and circumstances [28]. Moreover, each participant in the diagnostic process (including the patient, family, provider, and radiologist) compounds the effects of uncertainty. For example, patient and family expectations, based on their uncertainty about how the diagnostic process is unfolding, are a contributing factor in emergency physicians ordering self-acknowledged unnecessary imaging [29,30]. In conjunction, most imaging findings are not pathognomonic, and, as such, uncertainty is an intrinsic and ineradicable feature of radiology and medicine writ large [31].

"Therapeutic" Use of Diagnostic Imaging

In recent decades, the medical establishment has made efforts to better appreciate patients' values in clinical decision-making [32-34]. One patient-centered outcome is reducing the anxiety associated with diagnostic uncertainty, i.e., the "value of just knowing or finding out more, whatever the outcome" [35]. This concern has, however, engendered medically unnecessary imaging, referred to as 'therapeutic scans' [36]. In interviews with adult patients from primary care clinics, Suchsland et al. found that many patients desired imaging even if they thought it would likely not directly influence their health care [37]. One of the themes that emerged is the importance placed on testing for reassurance or 'peace of mind' [37]. Similarly, De Silva et al. found that reassurance was a common theme given by Australian general practitioners when deciding to order imaging [36]. They report often feeling compelled to comply with patient requests for imaging as a form of reassurance, even if they do not "necessarily agree with the nature of those requests" [36]. The costs and potential harms of imaging performed for emotional reassurance purposes, however, are substantial and must be considered within a value-based framework.

To study the effect of diagnostic tests on patients' worry about illness, anxiety, symptom persistence, and subsequent use of health care resources in patients with a low pretest probability of serious illness, Rolfe and Burton conducted a meta-analysis of 14 randomized controlled trials [38]. Importantly, they found that diagnostic tests for symptoms with a low risk of serious illness actually did very little to reassure patients, decrease their anxiety, or resolve symptoms either in the short (<3 months) or long term (>3 months). While these tests may reduce subsequent primary care visits, the number of patients needed to avoid one subsequent outpatient visit varied from 16 to 26, depending on the symptom [38]. This is not cost-effective, and as Dr. Kroneke noted in an associated commentary, 'If the cost of these expensive diagnostic tests is estimated conservatively to be $250 to $500 per test, $4000 to $16,000 would be expended to prevent a $100 primary care visit' [39].

Fear of Litigation

Heath notes that "when death or disease occurs prematurely and unpredictably, the linear rationality in the rhetoric of preventive medicine suggests that someone, somewhere, must somehow be at fault" [28]. This reductive, linear reasoning causes significant fear among practitioners, reduces their tolerance for uncertainty, and invites them to do more, rather than less [28]. This uncertainty often manifests as "defensive medicine" - the practice of medicine centered around self-protection from liability in the event of an injurious outcome [40]. In addition to undermining the doctor-patient relationship, defensive medicine also encourages the frequent use of low-value tests [40]. Regardless of whether these fears are proportional to the actual degree of risk practitioners face, defensive medicine is common and has been called an "epidemic," with some studies demonstrating that it is practiced more often than not [41,42]. Chen et al. found that 38% of CTs performed in trauma patients were ordered for "defensive purposes" - to protect the ordering physician from liability rather than furthering patient diagnosis [43]. Importantly, only 2.2% of patients who had CTs ordered out of fear of liability had changes in their management based on the findings [43]. In a 2017 survey of 2,106 physicians, Lyu et al. found that 65% believed that 15-30% of medical care (including tests) is unnecessary, and 85% of the cited reasons for this overtreatment were "fear of malpractice" [44].

Knowledge or Skill Deficits

Declining clinical acumen among healthcare providers may be an additional cause of overutilization and low-value imaging [45-47]. In fact, clinician focus groups acknowledge that overutilization may be, at least partially, related to a lack of confidence in their own clinical and physical examination skills [23].

Anecdotally, physicians' clinical diagnostic skills may have declined as their reliance on radiology has grown. In a study from the Netherlands, Erysodan et al. performed a retrospective analysis of 531 patients who underwent abdominal CT at a tertiary care center during on-call hours between 2005 and 2019. Clinical reasoning quality was expressed as a percentage (0-100%) of the difference between the differential diagnoses provided on the request form and the ultimate CT diagnosis. They found that the quality of clinical reasoning decreased significantly from 2005 to 2019 and was inversely proportional to CT volume [48], concluding that clinicians have become less skilled at clinical reasoning and therefore supplement their diagnostic deficiencies with imaging.

Incidental findings

Incidental findings, also called "incidentalomas," fuel overutilization and are often examples of "overdiagnosis," defined as a true-positive diagnosis that produces negligible or absent clinical benefit. Defined as "unanticipated imaging results unrelated to the patient’s chief concern" [49], incidental findings are increasingly common due to improvements in imaging technology, occurring in 15-30% of all diagnostic imaging tests and 20-40% of all CT and MRI examinations [49]. In fact, some studies find that incidental findings are diagnosed more frequently than the primary diagnostic entities for which the examinations were performed to evaluate [50,51].

In most cases, an incidental finding will not be associated with any high-risk history, signs, or symptoms because the imaging test was, by definition, performed for a different indication [49]. As noted by Davenport, "indolent disease will be detected more often than aggressive disease; and overdiagnosis and overtreatment will dominate - all while giving the illusion of improved care through early identification" [49].

These findings often lead to additional medical care, including further testing and treatment that may not benefit the patient but which exposes them to additional health risks [52]. In a survey of United States internists, Ganguli et al. found that 86.7% of 376 physicians reported that incidental findings caused their patients 'harm' - both psychological (68.4%) and physical (15.6%) [52].

It is important to note that incidental findings lead to low-value care because the patients in whom they are identified usually have a low pre-test probability of meaningful disease. Treatment of preclinical disease incidentally detected by imaging may not reduce mortality or morbidity but may paradoxically cause harm [49].

Unfortunately, however, it is often impossible to prospectively determine the likelihood of clinically significant disease in any specific patient at the time of incidentaloma identification - i.e., whether it will become symptomatic or remain indolent. Therefore, radiologists (and referring clinicians) often will not feel comfortable ignoring incidental findings, as the level of risk to any individual patient is uncertain [49]. Demonstrating a link between low-value care and incidental findings, 33.7% of ordering providers report that often the index test, which revealed an incidental finding, was itself not clinically appropriate [52].

Multidisciplinary culture change

The relationship between radiologists and their referring providers has changed dramatically over the past few decades. In the New York Times bestseller The Digital Doctor, Wachter notes, "When I was a medical student in the 1980s, the beating heart of the Hospital of the University of Pennsylvania was not the hospital’s mahogany-lined executive suite, nor the dazzling operating room [but was in] the decidedly unglamorous, dimly lit Chest Reading room..." In contrast, Wachter notes that by the mid-2010s, many internal medicine trainees barely knew the location of the radiology department [53]. Unfortunately, digital image archiving, along with economic pressures that emphasize efficiency and productivity at the expense of interaction, has led many radiologists and clinicians to minimize interaction with one another. In fact, some radiologists have come to think of clinical interactions as "distractions" [54]. The result of these trends is not only fewer opportunities for radiologists and clinicians to share information about patients but also lost opportunities for clinicians to clarify the meaning of radiology reports and avoid potential misunderstandings [55].

Solutions

Technological Solutions to Facilitate Communication

Technological advances, such as video teleconferencing, allow re-establishing "face-to-face" interactions between radiologists and referring providers on a case-by-case basis, such as during multidisciplinary tumor boards [56]. In general, such communication is desired, but the rising demand for radiologists to participate in multidisciplinary boards creates conflict because it is "uncompensated work" that takes time away from tasks for which they are paid [57]. In conjunction with these video meetings, integration of electronic medical records (EMR) into PACS can improve clinical care [58]. Access to this data has been found to positively influence clinical interpretation and medical management [59], but at the expense of radiologist "person-hours," which are increasingly in short supply in the current era of severe workforce shortages [59].

Although these technological solutions are helpful, they do not fully compensate for the loss of routine in-person communication between radiologists and caregivers, nor do they substitute for the clinician formulating a diagnostic hypothesis or even a coherent clinical question that imaging is meant to address. The importance of in-person communication between radiologists and clinicians outside the curated list of selected patients presented in most multidisciplinary meetings is nicely illustrated in Dickerson et al.'s review of 100 patients on an acute care surgery service with surgical attendings. These in-person meetings led to changes in the surgeons’ diagnostic impression and medical/surgical planning in 43% of cases despite the original written report containing all the necessary data [60].

In addition to large volumes of interventional, fluoroscopic, and ultrasound examinations, the annual workload of United States radiologists includes approximately 93 million CT examinations [8], >38 million MRI examinations (c.2016) [61], >13 million nuclear medicine studies (c.2016) [62], and >275 million X-ray procedures (c.2016) [62]. In 2010, Lin found that depending on the body organ system assessed, 43%-66% of CT examinations at one center were interpreted without consulting the electronic medical record [63]. Given increased imaging workloads since 2010, it is fair to posit that, in practice, the majority of radiology examinations are interpreted using only the often incomplete and incorrect clinical history provided, with resultant decreased interpretive accuracy [64]. In addition, it has been shown that problem lists and other summaries found in the patient's EMR are both frequently incomplete and incorrect [65].

Diagnostic Excellence/Diagnostic Stewardship

A meaningful way to reduce harm from overdiagnosis is to reduce the aggregate number of low-value studies performed. This is the essence of the "imaging stewardship" component of the Diagnostic Excellence initiative (11).

Unfortunately, efforts to curtail low-value diagnostic tests (such as the Choosing Wisely campaign initiated in 2012 by the American Board of Internal Medicine [66]) have had limited success [67].

Some authors have suggested that radiologists may ultimately need to act as 'gatekeepers' or 'stewards' of imaging to control overutilization [67-69]. Radiologist-driven gatekeeping is, however, challenging to effectuate in the United States secondary to workforce shortages, reimbursement incentives, interpersonal pressure, patient demand, low tolerance for uncertainty, radiologist unawareness of the patients’ clinical context, stigma attached to "rationing," and the perception of increased medicolegal risk [68]. Even so, radiologists may eventually be asked to perform regulatory roles in controlling high-end imaging utilization [70].

An additional means of reducing overutilization is improved communication with referring providers about the inherent limitations of imaging. Some authors note that imaging has come to be viewed as more of a laboratory test than a referral consultation [71] because clinicians frequently perceive it as 'objective.' Marquis et al. describe how published descriptions of imaging findings of disease entities, such as COVID-19, create a misconception that imaging findings are specific to a particular disease [72]. In a broad sense, this misconception leads clinicians to order non-indicated imaging tests in the hope of obtaining a specific diagnosis when that is often not possible.

Standardization of Recommendations and Nomenclature

Radiologists can help reduce diagnostic uncertainty by using standardized terminology and recommendations, as the current lack of standardization in reporting contributes to greater uncertainty, suboptimal clinical decision-making, compromised patient outcomes, higher costs, and more low-value care [73,74]. One successful effort at standardization has been the American College of Radiology's "Reporting and Data Systems" (RADS), which aims to reduce variability and ambiguity in reporting, promote effective communication between radiologists and referring providers, guide clinical management, and enable data-driven performance improvement [75]. Taking the lead from BI-RADS (Breast Imaging Reporting and Data System), there are an increasing number of standardization systems, such as TI-RADS, Lung-RADS, PI-RADS, CADRADS, LI-RADS, O-RADS, and C-RADS, developed to foster common lexicon and management recommendations [76-80]. Use of these systems can also aid radiologists in reporting findings in a manner that mitigates uncertainty and fear. Stewart et al. note that instead of reporting a 4 cm simple ovarian cyst in a postmenopausal woman and including the caveat 'cancer cannot be excluded,' radiologists can report 'Ovarian-Adnexal Reporting and Data System (O-RADS) category 2: this is a finding that is almost certainly benign (risk of important cancer less than 0.5%)' [9].

A recent paper from an Expert Panel of the ACR Commission on Quality and Safety proposed a potential framework for communicating the level of diagnostic certainty in radiology reports using specific terminology, which they referred to as the “2025 Certainty Scale,” using terms such as "Very likely" to indicate high, over 95% probability, "Equivocal" to indicate intermediate, 25% to75%, probability, and "Unlikely" to indicate low probability. If universally adopted, the group believes that the use of such a standardized lexicon would reduce the risk of errors resulting from clinicians’ misunderstanding the level of diagnostic certainty being reported by radiologists in their written reports [81].

Patient-Centered Outcomes

It is increasingly recognized that patient-centered outcomes are important. Like physical outcomes, psychological outcomes can be easily studied and evaluated [2]. Although early studies have strongly suggested that the temporary anxiolysis resulting from low-value imaging is not durable or cost-effective and can sometimes add to patient anxiety through detection of incidental findings, future research could further investigate in which situations the psychological value provided by seemingly low-value imaging is cost-effective. For example, a trial could be conducted in which alternatives to imaging, such as education, infographics, counseling, and usual care, are compared to imaging and assessed using standardized scales of anxiety [82] and total costs of care. This would help clinicians balance patient desires with the potential of iatrogenic harm from over-investigation [83].

Legal Initiatives

Federal legislators have demonstrated a commitment to rectifying the problem of low-value imaging. In 2014, the US Congress passed the Protecting Access to Medicare Act (PAMA), which set evidence-based criteria for reimbursement of advanced imaging under Medicare [84]. Beyond cost savings from reduced imaging [85], the act can improve patient outcomes by reducing radiation exposure and the sequelae of unnecessary imaging. The following year, Congress passed the Medicare Access and CHIP Reauthorization Act (MACRA), which adjusted Medicare reimbursement rates for covered medical services, including imaging, based on quality and cost-efficiency assessments [86]. In doing so, Medicare compensation is increasingly aligned with metrics of care value rather than volume.

Both laws, however, suffered from implementation problems with complexity and high cost of compliance being important impediments to effectiveness [87,88]. For example, the appropriate use criteria program (AUC) under the PAMA ambitiously planned to require clinicians seeking advanced imaging under Medicare to use an electronic interactive tool (Clinical Decision Support (CDS)) that communicates AUC information to radiologists [89]. CDS information would be provided to the Centers for Medicare and Medicaid Services (CMS) when claims are made for services provided. Subsequently, CMS would collect this information in real time to identify outlier clinicians, who would then be subject to prior authorization [90]. CMS, however, found that this reporting requirement was an “insurmountable barrier” to full implementation and paused the program, noting that if the “AUC program were implemented under the current system... <it> would pose a severe administrative and financial burden to providers and inevitably lead to inappropriately delayed or denied care to Medicare beneficiaries” [91]. Recently, however, senators have introduced a bill, the Radiology Outpatient Ordering Transmission (ROOT) Act, to simplify the AUC process and preserve the value-maximization efforts begun with PAMA [92]. If passed, there would be no real-time reporting requirement, and providers would instead attest that they reviewed the AUC at the point of care. Thereafter, CMS would conduct retrospective audits to ensure compliance and educate providers about value [92]. The use of AUC programs promises to both enhance diagnostic accuracy by guiding medical providers to choose appropriate imaging examinations and reduce unnecessary imaging.

Future considerations

Anticipated Persistence of the Supply/Demand Imbalance in Radiology

In sequential articles, Christensen et al. from the ACR Harvey Neiman Health Policy Institute estimated that by 2055, CT utilization is projected to grow by 25.1% and radiologist supply by 25.4%, assuming no growth in radiology residency positions [93,94]. As such, the current imbalance between imaging demand and radiologist supply is expected to remain essentially unchanged in the future.

These demand estimates are likely underestimated, as they do not account for the increased number of images per scan, a marker of imaging complexity [95]. Indeed, one study found that the number of images generated per month from 2009 to 2022 increased by 1,091%, translating into a 399% increase in the number of images available per full-time radiologist per month [95].

Potential Impact of Artificial Intelligence (AI) in Radiology Practice

Recent AI-based techniques also hold promise for increasing diagnostic accuracy, but they may also engender the proliferation of low-value imaging. For example, “opportunistic imaging” is a form of AI-based screening performed on an undefined population without specific disease risk (i.e., anyone undergoing advanced imaging for any reason, from which biomarkers can be generated) [96]. Given that the post-test probability of disease is highly dependent on the pretest probability, screening low-risk patients with sensitive imaging modalities such as CT and MRI will be burdened by a high false-positive rate, thereby promoting low-value care [96]. In addition, AI-generated prognostic algorithms will almost certainly increase imaging utilization. Imagine an asymptomatic 55-year-old woman who undergoes annual mammography for breast cancer, the report for which concludes “Negative, but high risk of breast cancer” [96]. Without available laboratory-based markers to monitor for emergent disease, the most common logical approach in clinical scenarios where symptoms only occur late in disease (e.g., lung cancer) is to obtain additional or more frequent imaging, with attendant costs and potential risks of harm [96].

Another potential driver of low-value imaging is automation bias, a form of cognitive bias that occurs when humans favor decisions generated by machines, often disregarding human decisions or contrary data [97]. It has been suggested that this bias may be more common under conditions of elevated workloads and exacerbated by operator inexperience [97]. Dratsch et al. found that inexperienced radiologists were significantly more likely to follow suggestions of AI when it incorrectly suggested a higher BI-RADS category [98].

Bernstein et al. found that when AI provided incorrect results, radiologists had higher false-negative and false-positive rates; however, this effect was mitigated when radiologists believed AI data would be deleted from the patient's file. In short, radiologists were less likely to disagree with AI (even when incorrect) if there is a record of that disagreement [99]. When AI reports that a study is potentially positive, Banja et al. note that radiologists may feel anxiety rejecting its recommendations, given the potential scenario wherein a patient experiences a poor outcome from this rejection and alleges negligence [100]. The ‘safe option’ from a discovery and liability standpoint is to agree with AI when its results are ‘on record’ [101].

Using clinical vignettes, Bernstein et al. found that this liability concern is well-justified, as survey participants were more likely to side with plaintiffs, finding that a radiologist who failed to detect an abnormality was more culpable when they used an AI system that detected the abnormality [102].

Best practices in the use of AI as an adjunct to imaging will need to include informing patients about the use of AI tools for their care while simultaneously minimizing unintended negative consequences [103].

Potential Radiation Risks From Inappropriate Use of CT

With regard to the population risk for radiation exposure, Smith-Bindman et al. used a risk model to project the number of future lifetime cancers from CT scans [8]. Using utilization data from 2023 (93 million CT scans), this model found that CT imaging will result in 102,700 additional projected lifetime cancers, 5% of all new cancer diagnoses annually [8]. They note that this ’would place CT on par with other significant risk factors, such as alcohol consumption (5.4%) and excess body weight (7.6%)’ [8] and further note that this preponderance of CT imaging was partially secondary to ‘growth in low-value potentially unnecessary imaging’ (italics added) [8]. Although there is inherent uncertainty and controversy regarding these cancer risk estimates, an accompanying editorial notes the importance of interventions to reduce low-value imaging, including diagnostic algorithms and educational interventions [104].

## Conclusions

The traditional primary function of imaging was to reduce referring providers' diagnostic uncertainty and aid management decisions. Historically, imaging was applied judiciously when needed to answer a specific clinical question. This deliberate, considered approach to imaging use had a high likelihood of leading to high-value care. In recent years, however, the threshold for ordering imaging has been significantly lowered, leading to far less focused and increasingly indiscriminate imaging. As such, radiologists are often asked to extract information from examinations with essentially no or incompletely established pre-test probabilities of disease. As examinations are increasingly performed on a lower-risk, more general population, the result is a higher prevalence of low-value care. These trends effectively constitute a substantial “mission creep” for the practice of radiology, whereby imaging has increasingly become the primary means of initial patient assessment, essentially replacing the basic history and physical examination, a function for which it is poorly suited.

Overutilization of imaging results in overdiagnosis, overtreatment, rising costs, harm from incidental findings, and the erosion of radiology’s value proposition. In addition, technological trends, including emergent AI-based techniques, suggest that increasing amounts of low-value imaging are likely to be performed in the future as clinicians and radiologists attempt to manage clinical uncertainty, reduce liability, and mitigate risk from an ever-growing and increasingly probabilistic data stream. In contrast, the principles of diagnostic excellence and diagnostic stewardship, if operationalized effectively, can curb these trends toward overutilization. Given the widening mismatch between imaging capacity and demand, economic imperatives to maximize the value of healthcare services, and the ethical imperative to "do no harm," the time to act decisively and "bend the curve" on low-value imaging is now.
